# Recent Progresses in Adsorption Mechanism, Architectures, Electrode Materials and Applications for Advanced Electrosorption System: A Review

**DOI:** 10.3390/polym14152985

**Published:** 2022-07-23

**Authors:** Youliang Cheng, Jiayu Shi, Qingling Zhang, Changqing Fang, Jing Chen, Fengjuan Li

**Affiliations:** 1 Faculty of Printing, Packaging Engineering and Digital Media Technology, Xi’an University of Technology, Xi’an 710048, China; chengyouliang@xaut.edu.cn (Y.C.); 15595365131@163.com (J.S.); zhangqingling0812@163.com (Q.Z.); chenjing@xaut.edu.cn (J.C.); 2 School of Mechanical and Electrical Engineering, Xinjiang Institute of Technology, Aksu 843000, China; fengjuan_1984@163.com

**Keywords:** electrosorption, water treatment, adsorption mechanism, architecture, electrode materials

## Abstract

As an advanced strategy for water treatment, electrosorb technology has attracted extensive attention in the fields of seawater desalination and water pollution treatment due to the advantages of low consumption, environmental protection, simplicity and easy regeneration. In this work, the related adsorption mechanism, primary architectures, electrode materials, and applications of different electrosorption systems were reviewed. In addition, the developments for advanced electrosorb technology were also summarized and prospected.

## 1. Introduction

As the economy develops rapidly, a large number of pollutants are discharged into the water environment, resulting in the serious pollution of locally available freshwater resources [1,2]. Globally, water pollution has become a hot topic in recent years. In order to address this issue, chemical precipitation, membrane separation, ion exchange, distillation, adsorption and other technologies have been proposed by researchers [3,4,5,6,7,8,9,10]. In general, ordinary adsorbents have been widely used in the field of water treatment due to the simple operation process. Its basic adsorption principle is the mass transfer process in which the adsorbates are transferred from the liquid phase to the surface of the adsorbents combined by physical and/or chemical actions. However, most of adsorbents are limited in the practical application because of secondary pollution, low recovery and/or low adsorption efficiency, whereas electrosorb technology (EST), also called capacitance deionization technology (CDI) based on ion electrosorption, has attracted much attention in the field of seawater desalination and water pollution treatment due to the high efficiency, low energy consumption, environmental protection, and simple regeneration process [11,12,13,14], which is regarded as a promising strategy for water treatment. To select a proper strategy, it is necessary to discuss the adsorption mechanism, primary architectures, electrode materials, and applications for different electrosorption systems.

## 2. Adsorption Mechanism and Cell Architectures of CDI

### 2.1. Adsorption Mechanism

For CDI, the adsorption mechanism is based on the electrochemical double layer (EDL) with capacitive characteristics. During the process of electrosorption for wastewater, after applying an external voltage (the voltage ≤ 2 V for preventing the electrolysis of water), the surface of the adsorption electrode is charged, and then an EDL is formed between electrode materials and the solution. Positive and negative ions in the water are attracted to the cathode and anode, respectively, which are stored by the electrodes of EDL. When the voltage between the two electrodes is stopped or applied in reverse, the desorption process will begin [15,16,17,18]. During the process of desorption, acid, alkali, and other reagents can be greatly reduced by post-treatment, thus avoiding secondary pollution [19,20]. In addition, the direct voltage applied between the electrodes just drives the adsorption, and then there are a few other side reactions such as water electrolysis and ion precipitation. Electrode materials also can be easily recycled and regenerated due to the adsorption and desorption of ions that occurs during the cyclic charging and discharging operation of EDL [21].

**Figure 1:**
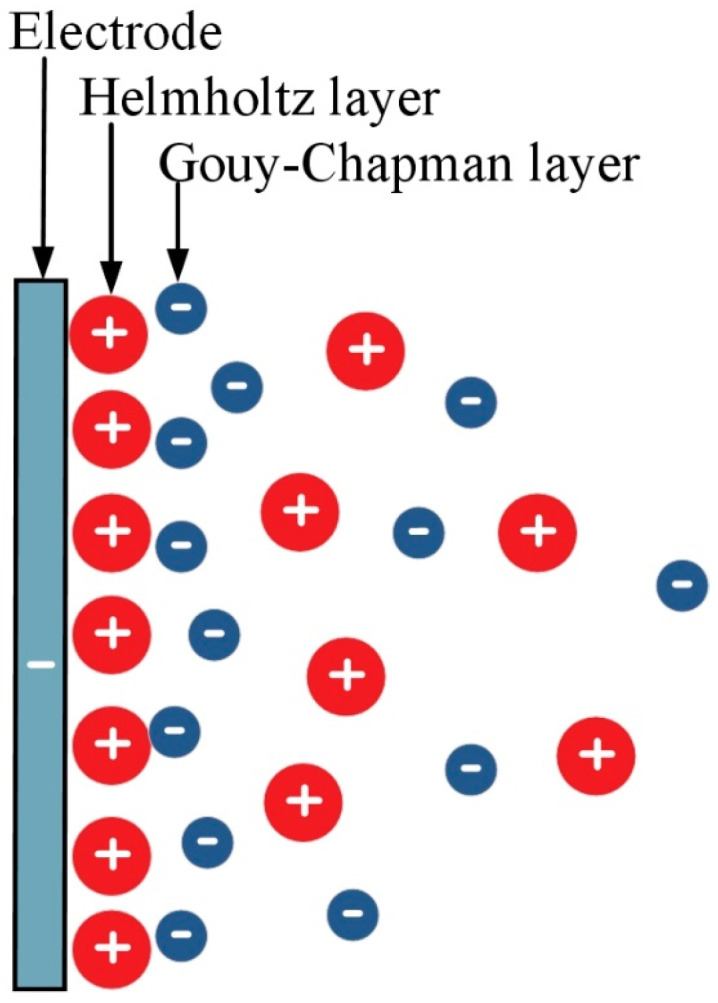
Schematic diagram of Gouy–Chapman–Stern model.

For the ideal adsorption process, there is no electron transfer on the solid–liquid interface between the electrode and the solution, and it is a non-Faraday process with low energy consumption [21]. In addition to the dominant electric adsorption, this process also involves physical adsorption and chemical adsorption [22,23]. Therefore, the Gouy–Chapman–Stern double layer model (Figure 1), including the inner layer (Helmholtz layer) near the electrode side and the diffusion layer (Gouy–Chapman layer) near the solution side, is widely accepted to explain the distribution of adsorbates in these double layers. The distance between the boundary of the inner layer and the electrode surface is about 1 nm, and the ion concentration for the diffusion layer is lowered where it is farther from the electrode surface [11,24,25,26]. Furthermore, the total electrode surface capacitance, *C_T_*, can be calculated according to Equation (1):

(1) $$\frac{1}{C_{T}}=\frac{1}{C_{M-H}}+\frac{1}{C_{H-S}}$$

where *C_M−H_* is the capacitance of the Helmholtz layer, *M* is the position of the ion closest to the electrode surface, *H* is the outermost position of the Helmholtz layer, *C_H−S_* is the capacitance of the Gouy–Chapman layer.

Due to inapplicable conditions of high ion concentration and hydrated ions, some developments to the Gouy–Chapman–Stern double layer model have been made by researchers. In 1947, the Grahame model further refined the Stern layer into the inner Helmholtz plane (IHP) and outer Helmholtz plane (OHP) based on hydration and unhydration of adsorbed ions [27]. According to the classical double layer theory and the characteristics of ionic liquids, established mean-field theory (MFT) by Kornyshev and co-workers can be used to explain the differential capacitance and double layer structure of the electrolyte/electrode interface. This theory can make up for the limitations of the Gouy–Chapman–Stern model and point out the differential capacitance change from a “bell-shaped” to a “camel-shaped” curve with the decreasing of the system compression rate [28,29,30]. However, due to different types of ionic liquids, the basic principles of electrosorption and the characteristics for the migration and diffusion of ions in micropores need to be explored furtherly.

### 2.2. Cell Architectures of CDI

**Figure 2:**
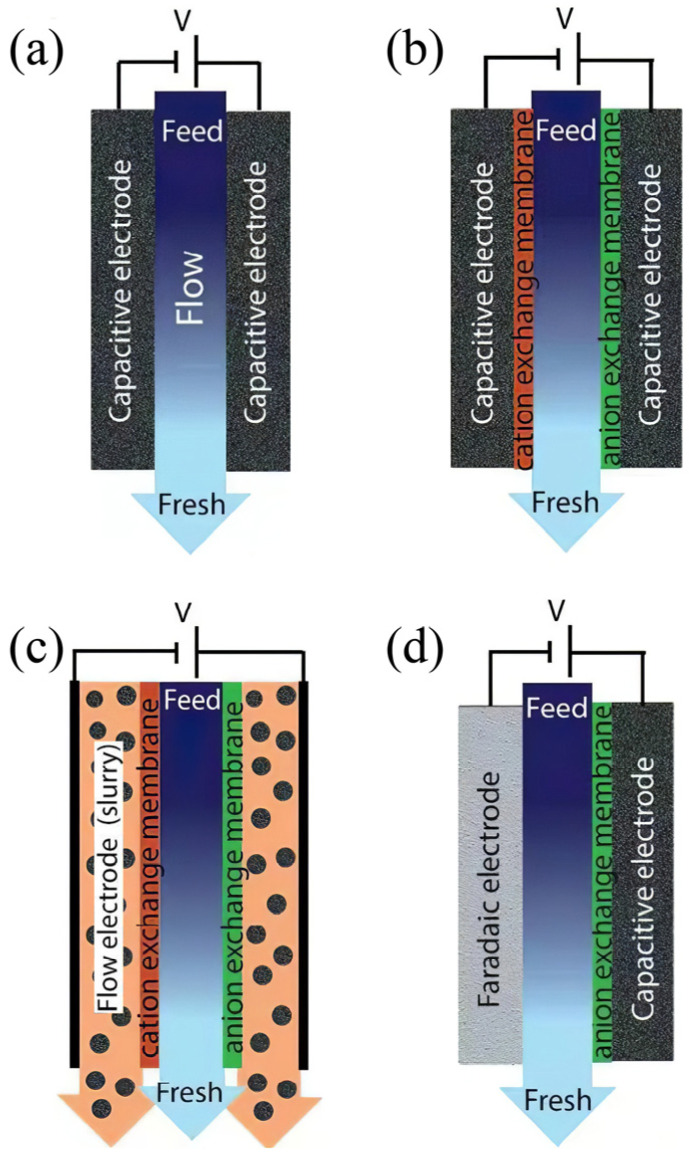
Schematic diagrams of (**a**) traditional CDI cell, (**b**) MCDI cell, (**c**) FCDI cell, (**d**) HCDI cell. Reprinted from Reference [33] with permission.

To our knowledge, the ion removal efficiency of CDI technology can be improved by optimizing the device configuration and performances of electrode materials. The traditional CDI system (Figure 2a) has some inherent obstacles, including co-ion effect interference, intermittent operation of adsorption and desorption, and oxidation of electrode materials [31,32,33]. To overcome the shortcomings, new CDI cell architectures have been developed such as membrane capacitance deionization technology (MCDI), flow electrode capacitance deionization technology (FCDI), and hybrid capacitance deionization technology (HCDI), which can introduce novel features into this field.

#### 2.2.1. Membrane Capacitive Deionization

**Figure 3:**
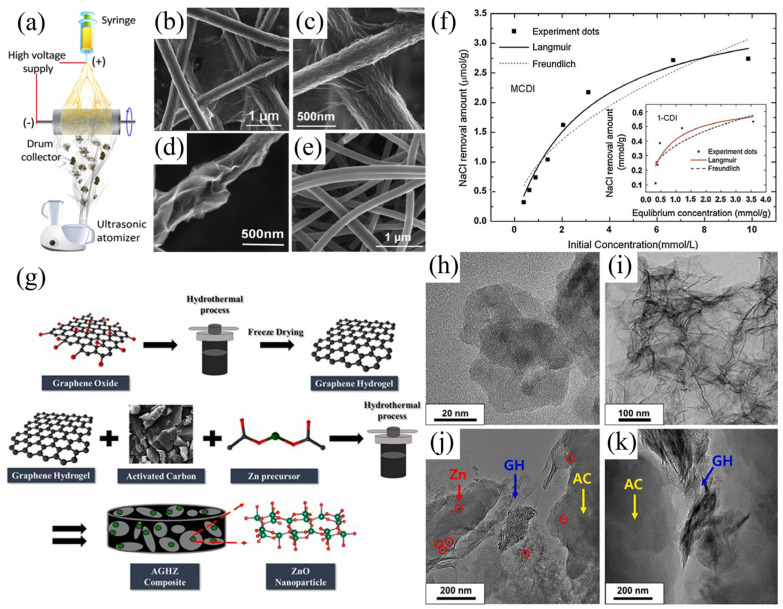
(**a**) The preparation illustrating diagram of electrostatic spinning and ultrasonic spraying process; (**b**) SEM image of RGO/ACF composite webs; (**c**,**d**) SEM images of the individual S −RGO/ACF fiber; (**e**) SEM image of ACF surface. Reprinted from Reference [65] with permission. (**f**) Adsorption isotherms for MCDI and CDI systems. Reprinted from Reference [37] with permission. (**g**) The preparation illustrating diagram of AGHZ nanocomposites, (**h**) TEM image of AC, (**i**) TEM image of GH (ZnO nanoparticle-incorporated graphene hydrogel), (**j**,**k**) TEM images of AGHZ. Reprinted from Reference [76] with permission.

In 2006, Lee and colleagues proposed MCDI, which integrates ion exchange membranes into CDI cell electrodes [32]. For the MCDI cell (Figure 2b), the cation exchange membrane (CEM) and the anion-exchange membrane (AEM) are attached to the surface of the cathode and anode, respectively. The ion exchange membranes can assist to separate positive and negative charged ions, and the co-ions can be effectively prevented from the adsorption onto the electrodes while the counter-ions are adsorbed sufficiently by the electrodes [34,35]. In addition, when applying a reverse voltage to the electrode for the regeneration, the ion exchange membrane can effectively prevent the opposite ions from adsorbing to the electrode, which solves the problem of incomplete regeneration of CDI electrode and improves the ion storage [31,32]. Therefore, the removal efficiency of MCDI is higher than that of CDI [36]. Carbon nanotubes and nanofibers (CNTs-CNFs) composites were used as CDI electrodes by Li et al., and the desalting efficiency of MCDI was 49.2% higher than that of CDI (Figure 3f) [37]. In addition, Lee et al. applied the voltage of −0.5 V for MCDI desorption and found that the desorption degree reached 80% after 130 s, while the short-circuit desorption process needed 150 s when reaching the same desorption degree. Due to the electrostatic repulsion between the adsorbent ion and the electrode with the same charge, the application of a reverse voltage could accelerate the desorption of MCDI [38]. Biesheuvel et al. investigated the ion diffusion characteristics on the membrane surface and electrode surface and found that the mass transfer resistance of the retention diffusion layer and ion exchange membrane is the main factor affecting ion diffusion during the MCDI desalination process [39]. However, high resistance, high energy consumption, and limited surface area for ion adsorption restrict the application of MCDI architecture in the field of capacitive deionization [40].

#### 2.2.2. Flow Capacitive Deionization

The electrosorption processes of conventional CDI and MCDI are not continuous because the electrodes need to be regenerated when they are saturated, which increases the complexity of the operation. In addition, charge/discharge conversion could lead to a high energy consumption [40,41,42,43]. To solve the problems of fixed-electrode CDI technology, FCDI was presented by Jeon and co-workers (Figure 2c) in 2013, and the tiny modification of MCDI with carbon suspension as flow electrodes was carried out [44]. The ions in the solution migrate to the two electrodes under an applied voltage and enter the flow electrodes through the ion exchange membrane. Because of the fluidity of the slurry, the system creates a continuous adsorption process inside the cell and the desorption occurs outside the cell. Compared with fixed-electrode CDI, the FCDI system shows continuous ion removal behaviors and can handle the solution with a high concentration of ions [45,46,47]. Nevertheless, the current efficiency of FCDI is lower than that of CDI/MCDI because the water composition of the feed electrode as weak electrolyte is unfavorable for the charge transfer from the collector to the slurry. Additionally, massive deionization of FCDI is greatly constrained by the poor conductivity of flow electrode slurry and the risk of channel blockage. In order to improve the desalination efficiency of FCDI system, Huang’s group used anion/cation compounds as conductive dispersants doped with activated carbons (AC) and then as flow electrodes to construct an efficient desalination system. Among them, the utilization and dosage of AC were improved by electrostatic repulsion of anion/cation compounds and then reduced co-ion effect in electrode slurry [48]. In addition, to break the limitation of FCDI working voltage by water decomposition potential (1.23 V) in practical application, a novel asymmetric FCDI (AFCDI) device, first proposed by Xu’s group, used AC/manganese dioxide suspension as a positive pole and AC suspension as a negative pole. The system increased the operating voltage to 1.8 V by enlarging the potential window between positive and negative electrodes, in which the potential window range of positive and negative electrodes was 0–1.0 V and −0.8–0.2 V, respectively. Thus, the potential on the surface of the positive and negative electrodes is lower than 1.23 V, which effectively avoids hydrolysis under high pressure. In addition, the system further increased the desalination rate of FCDI by 19% compared with the conventional condition of 1.2 V [42]. However, FCDI is still far from practical application for now. The complex configuration and the high requirement for sealing FCDI devices make the operation cost high. In addition, low charge transfer and low feed solution velocity also need to be solved [44].

#### 2.2.3. Hybrid Capacitive Deionization

MCDI can slow down the undesired faradaic reactions, and thus improve the cycling performance of carbon electrodes by introducing ion-exchange membranes (IEMs) [49,50,51,52,53,54,55,56]. Nevertheless, the high cost of IEMs essentially limits the practical application. Therefore, the HCDI system was proposed by J. Lee and co-workers in 2014, which can undergo an ion-selective removal process and promote charge efficiency without IEMs [57]. Moreover, this system improves the ion removal efficiency and keeps good reversibility and cycle performance [46]. As shown in Figure 2d, an asymmetric system of HCDI normally consists of a battery electrode and a capacitive electrode [33,58]. Zhao’s group developed a membrane-free HCDI system by using amino-functionalized commercial AC and Fe_3_O_4_/PGCN as the anode and the cathode, respectively, which had good regenerative properties, high cyclic stability, and the removal efficiency of 90%~97% for the mixture of Mg^2+^, Zn^2+^, Cu^2+^, Cd^2+^ and Pb^2+^ [59]. Chen’s group designed NTP/MXene (NTP/M) nanohybrid as a Faraday electrode for HCDI. Due to the high conductivity and sodium ion insertion of the HCDI system, the maximum desalination rate even reached 128.6 mg/g, while the desalination capacity of conventional CDI technology is less than 20 mg/g [60]. Although the HCDI system has a higher desalination capacity than CDI, the desalting capacity of some Faraday electrode materials tends to decay rapidly during the cycle of adsorption and desorption. Therefore, Faraday electrode materials with high cycle performance for HCDI also need to be further explored [46,61].

## 3. Electrode Materials of CDI

The electrode is the key component of electrosorption technology. Commonly, the ideal electrode materials should have the following characteristics: high specific surface area, good hydrophilicity, suitable pore size, good electrical conductivity, and electrochemical stability for high double electric layer capacitance, fast charge transfer, and long service life [11,17,21,62]. Carbon materials usually have a high specific surface area, stable porous structure, and excellent electrical conductivity, well meeting the comprehensive requirements of CDI electrode materials. Due to the low adsorption capacity of single carbon material, some researchers attempt to combine carbon material with other carbon materials, metal oxides, and/or conductive polymers to form carbon-based composites and improve the electrosorption capacity of the primary carbon materials.

### 3.1. Carbon-Carbon Composite Materials

To date, carbon materials including graphene, AC, carbon fiber, carbon aerogel, CNTs and so on, can be used as CDI electrodes. However, the CDI adsorption capacity and electrochemical performance of single carbon electrodes are greatly limited due to the low charge capacity. Therefore, in view of the deficiency of a single form of carbon material, a variety of carbon materials can be combined to obtain carbon–carbon composite electrode materials. The carbon–carbon composites have a synergistic effect, and their CDI performance is better than that of the single composition material [63,64].

Due to the negative effect of polymer binders on the electric adsorption capacity of carbon material powders as CDI electrodes, monolithic carbon materials with free binders as CDI electrodes have been a concern of researchers. Wang’s group fabricated monolithic web electrodes composed of activated carbon nanofiber/reduced graphene oxide (ACF/rGO) (Figure 3a–e). To prevent rGO sheets from restacking, freestanding monolithic carbon nanofiber webs were used as a frame and the rGO network facilitated rapid electron transfer through the matrix and efficient storage of ions, presenting a desalination capacity of 9.2 mg/g under the conditions of 100 mg/L NaCl and 1.2 V applied voltage [65]. Cao et al. prepared three-dimensional (3D) graphene hydrogels by the one-step method which were modified with single-walled carbon nanotubes (SWCNTs) or multi-walled carbon nanotubes (MWCNTs) for increasing the electrical conductivity and reducing the graphene sheets aggregation. Moreover, as-prepared 3D graphene hydrogels as free-standing electrodes alleviated the blocking by binders, and the adsorption capacity of SWCNTs/rGO (48.73 mg/g) was higher than MWCNTs/rGO under the conditions of 300 mg/L NaCl and 2 V applied voltage [66]. To improve the electric properties of activated carbon cloth (ACC) webs, Liu’s group fabricated ACC/GO electrodes by the vacuum filtration method for removing Co^2+^ and Cs^+^, thus ion-transport obstacle was reduced and the electrosorption capacity increased compared with ACC [67].

For the electrodes composed of powdery carbon–carbon composites, the polymer binder may increase the resistance, block some pores, and have a shielding effect on the effective sites of the electrode surface, resulting in the reduction of the electric adsorption capacity. Therefore, the carbon–carbon composite electrode with free binders is an alternative for the CDI system.

### 3.2. Metal-Oxide and Carbon Composite Materials

Metal-oxides and carbon composite materials as CDI electrodes have been developed in recent years, due to their high desalination capacity and excellent cycling stability. Mixing metal oxide nanoparticles with carbon-based materials can improve the specific capacitance and hydrophilicity of electrodes, prevent the accumulation and blockage of active substances in electrodes, and inhibit the physical adsorption of the electrode [68,69,70,71,72,73,74,75]. Some metal oxides such as MnO_2_, ZnO, and Fe_3_O_4_ have been widely used in hybrid electrodes through in situ growth method, hydrothermal method, precipitation method, and so on.

AC electrode material has a low cost and good physicochemical properties, but the poor wettability limits its application. To address this issue, ZnO nanoparticle-incorporated AC/graphene hydrogel composites (AGHZ) were prepared by Ahmed S. Yasin’s group (Figure 3g–k). The wettability and conductivity of AGHZ increased significantly compared with AC and ZnO nanoparticle-incorporated graphene hydrogel (GH), and the adsorption capacity of AGHZ reached 9.95 mg/g under the conditions of 50 mg/L NaCl, and 1.2 V applied voltage [76]. In addition, the modification of AC/ACC electrodes by using various metal oxides can improve the charge storage. For example, Htet Kyaw et al. prepared SiO_2_ nanoparticles decorated ACC electrode via in situ deposition as the CDI electrode and found that the local electric field around the SiO_2_ NPs dielectric coating was enhanced and this modification protected the electrode surface from oxidation [77].

**Figure 4:**
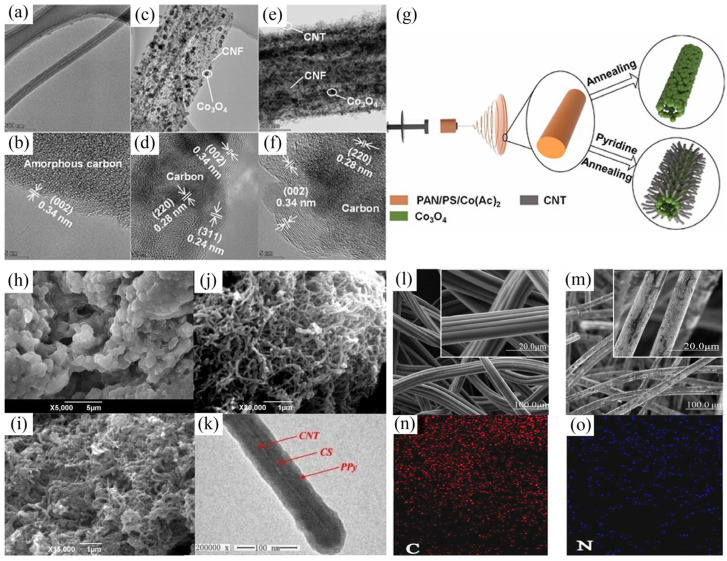
TEM images of as-prepared CNF (**a**,**b**), Co_3_O_4_@CNF (**c**,**d**) and Co_3_O_4_@CNF@CNT (**e**,**f**); (**g**) the preparation schematic of Co_3_O_4_@CNF and Co_3_O_4_@CNF@CNT. Reprinted from Reference [79] with permission. Morphologies of PPy/CS (**h**), PPy/CNT (**i**) and PPy/CS/CNT (**j**,**k**). Reprinted from Reference [86] with permission. Morphologies of ACF (**l**) and ACF/PANI (**m**), element mapping of ACF/PANI (**n**,**o**). Reprinted from Reference [87] with permission.

In addition, Co_3_O_4_ particles also attracted extensive attention from researchers due to the high theoretical capacitance and wide usability. Nevertheless, the unsuitable bulk structure, poor electrical conductivity, and cycling stability for Co_3_O_4_ hinder its commercial application. Divyapriya et al. designed graphene/Co_3_O_4_ composites by using graphene as a support matrix and applied it to the HCDI system for seawater desalination. Although carbon coating improves desalination performance to a certain extent, the graphene/Co_3_O_4_ composite still has the problems of non-porous and uneven structure [78]. Thus, a free-standing cathode (3D flexible hierarchical Co_3_O_4_/CNTs decorated hollow nanofiber film) for HCDI system was prepared by Guo’s group using electrospinning (Figure 4a–g). The self-supporting of the electrode eliminates the need for polymer binders, which do not contribute to CDI performance, simplifying the electrode manufacturing process. It was noted that the adsorption capacity of this system reached to 58.6 mg/g under the conditions of 1500 mg/L NaCl and 1.4 V applied voltage [79].

These metal-oxide and carbon composite materials have an application prospect. However, it should be noted that some metal oxides will precipitate out under acidic conditions, which will reduce the conductivity of the solution and cause secondary pollution. Thus, this issue for composite electrodes is also needed to be addressed in future research.

### 3.3. Conductive Polymers and Carbon Composite Materials

Conductive polymers with high molecular weight have exhibited the characteristics of both polymer and metal materials [3,4,5], which can be used as a promising candidate material for electrodes. Common conductive polymers include polyaniline (PANI), polypyrrole (PPy), and polythiophene [80,81]. It is noted that the mechanical properties of the conductive polymer are poor due to the contraction of the skeleton and expansion of the chain during the cycling of charging and discharging, leading to poor electrochemical stability. Therefore, many efforts have been made to improve the stability and electrochemical property by mixing conducting polymers with carbon materials. PANI has a high content of nitrogen and a similar structure to graphite, and then the electrical adsorption is improved by amine and imine functional groups [82,83,84,85]. Considering these conditions, 3D PANI nanotubes (PaniT) were prepared by Nie’s group via chemical polymerization of sulfonated polystyrene (PS) nanofibers and following removal of PS template, and then PaniT-AC composite electrodes were obtained by using PaniT as additives [85]. When the inner diameter of PaniT was 20 nm, the maximum deionizing capacity of the NaCl solution was as high as 30.5 mg/g compared with AC//AC capacitor (11.0 mg/g) under the conditions of 500 mg/dm^3^ NaCl and 1.72 V applied voltage.

At present, the commonly prepared routes for conducting polymer and carbon composites include the melt blending method, in situ polymerization, and hydrothermal method. Zhang’s group utilized in situ polymerization to fabricate polypyrrole (PPy)/chitosan/CNT composite electrode (Figure 4h–k), and the adsorption capacity (16.83 mg/g) for Cu^2+^ under the conditions of 100 mg/L CuSO_4_ and 0.8 V applied voltage was 2.1 times of that for PPy/CNT composite electrode [86]. Conductive ACF has high chemical stability, conductivity, and specific surface area. Nevertheless, its adsorption capacity is low, leading to a significantly lower CDI capacitance. In addition, PANI-modified commercial ACF with a low cost could have a high electric adsorption capacitance. Therefore, Tian’s group utilized in situ electrochemical polymerization to fabricate PANI-decorated ACF electrode (Figure 4l–o), and the maximum adsorption capacity under the conditions of 200 mg/L NaCl and 2 V applied voltage reached 19.9 mg/g. In addition, the ACF/PANI electrode had a higher capacitance and lower charge transfer resistance (1.17 Ω) compared with the ACF electrode (1.69 Ω) [87].Additionally, Sun’s group utilized the one-step hydrothermal method to load WO_3_ and PPy to ACF as cathode, and then made up an electrochemical system with ACF as the anode [88]. The adsorption degree of this system for Cu^2+^ and citric acid (CA) reached 97.8% and 80.1% (in 5 h), respectively.

Up to now, the application of conductive polymers and carbon composite materials in electric adsorption is still in the initial stage, the poor mechanical property and chemical stability of conductive polymers are the main obstacles which are also needed to be solved in future research.

## 4. Applications of Electrosorption Technology in Water Treatment

The salt removal from water by using electrosorption has been studied since 1960s, and the application of this technology began in the mid-1990s when Lawrence Livermore National Laboratory developed the first set of electrosorption application devices in 1996 [89]. Nowadays, this technology has been widely used in the water treatment, such as seawater desalination, removal of heavy metal ions and other harmful ions in wastewater, removal of a variety of organic compounds in wastewater and so on.

### 4.1. Removal of Salt Ions from Water by Electrosorption

To ensure the safety of industrial and domestic water supply, desalination technology has attracted the attention of many researchers. This concept of “electrochemical desalination” was put forward by Evans and Hamilton [90]. Compared with reverse osmosis (RO) and multi-stage flash (MSF), the electrosorption desalination technology shows low energy consumption, easy operation, and so on. To improve the electrosorption desalination ability of graphene, Ahmed’s group prepared graphene/SnO2 NPs composite materials by microwave method as CDI electrodes, and this ability was significantly better than that of original graphene [91]. In addition, Zhang et al. prepared graphene/carbon aerogels (GCCAs), which achieved the best salt adsorption capacity (SAC) of 26.9 mg/g under the condition of 500 mg/L NaCl [92]. Furthermore, carbon aerogels were also prepared by carbonizing the composite of PANI and GO, whose adsorption capacity was 15.7 mg/g under the conditions of 500 mg/L NaCl and 1.2 V applied voltage [93]. Wang’s group prepared graphite porous carbon nanosheets (GPCSs) by activating and graphitizing straw waste as the electrode material, and the electrosorption capacity of the sample for 500 mg/L NaCl solution is 19.3 mg/g when 1.2 V voltage was applied [94]. Due to the low cost of biomass derivatives, Lu’s team synthesized porous carbon nanoflakes (PCNs) by using xylose as the carbon source through the carbonization, and the maximum SAC of PCNS CDI electrode for 1000 mg/L NaCl solution reached to 16.29 mg/g when the voltage of 1.2 V was applied [95]. Additionally, Li et al. obtained phosphorus (P)-doped carbon nanofiber aerogel (P-CNFA) by using bacterial cellulose as the raw material via freeze-drying and heat treatment, and the SAC of P-CNFA reached 16.20 mg/g for 1000 mg/L NaCl solution under working voltage of −1.2 V [96]. Therefore, we can infer that salt ions will be removed efficiently via electrosorption technology.

### 4.2. Removal of Heavy Metal Ions and Other Harmful Ions from Wastewater by Electrosorption

The rapid development of industry leads to the increasing water pollution caused by heavy metals, which not only poisons aquatic organisms in water, but also endangers human health. The traditional treatment methods for heavy metals in wastewater mainly include chemical precipitation, coagulation–flocculation, flotation, ion exchange, membrane filtration and adsorption [97]. However, the above methods have some limitations. In 1997, Farmer et al. utilized the capacitive method to remove Cr^6+^ efficiently [98]. Subsequently, Oda’s group investigated the removal effect of Cu^2+^ and Zn^2+^ by using AC electrode, and then successfully introduced CDI technology into the field of heavy metal ion removal [99]. To research the influences of surface modification on the capacity of ACF cloth for heavy metal ions adsorption/electrosorption, Huang et al. conducted the adsorption and electrosorption of Cu^2+^ in wastewater by using different modified ACF cloth electrodes [100]. The results showed that the removal degree for the electrosorption was 2.2 times higher than that for the adsorption. In addition, Dai’s group used AC as an electrode for the electrosorption of As (III) in an aqueous solution, and found that the electrosorption capacity increased with the increase in voltage, initial As (III) concentration, and pH [101]. Furthermore, Huang’s group investigated the removal rate of Cd^2+^, Pb^2+^, and Cr^3+^ as well as the mixture by using CDI system and found that the electrosorption can effectively remove these metal ions and the removal rate was positively correlated with the applied voltage [102]. To our knowledge, MnO_2_/carbon composites have a high adsorption capacity for heavy metal ions in wastewater. Thus, Hu’s group prepared MnO_2_/CF composite materials via an electroplating method as the electrical adsorption electrode [103]. The adsorption capacity of Cu^2+^ for MnO_2_/CF composite electrode reached 172.88 mg/g under a working voltage of 0.8 V, which was more than two times for ordinary MnO_2_ adsorbent in the absence of an electric field. Moreover, Liu and co-workers fabricated activated carbon cloth/graphene oxide composite (ACC/GO) by vacuum filtration process, which was used as the CDI electrode to remove Co^2+^ and Cs^+^ in water. When 1.2 V voltage was applied to the composite electrode, the maximum adsorption capacity of Co^2+^ and Cs^+^ can reach 16.7 mg/g and 22.9 mg/g, respectively, under the condition of the CoCl_2_ solution concentration and CsCl solution concentration of 20 mg/L [67]. Due to the low cost and high capacitance of MnO_2_, Li’s group synthesized α-MnO_2_ nanoparticles by the hydrothermal method, combined with carbon fiber paper (CFP), to obtain α-MnO_2_/CFP as CDI electrode material [104]. The results show that the removal capacity of nickel ion for the composite reached 16.4 mg/g more than twice that for activated carbon under the same electrosorption conditions. Therefore, the electrosorption behaviors for different metal ions and harmful ions by the CDI system exhibit an obvious difference.

### 4.3. Removal of Various Organic Compounds from Wastewater by Electrosorption

**Figure 5:**
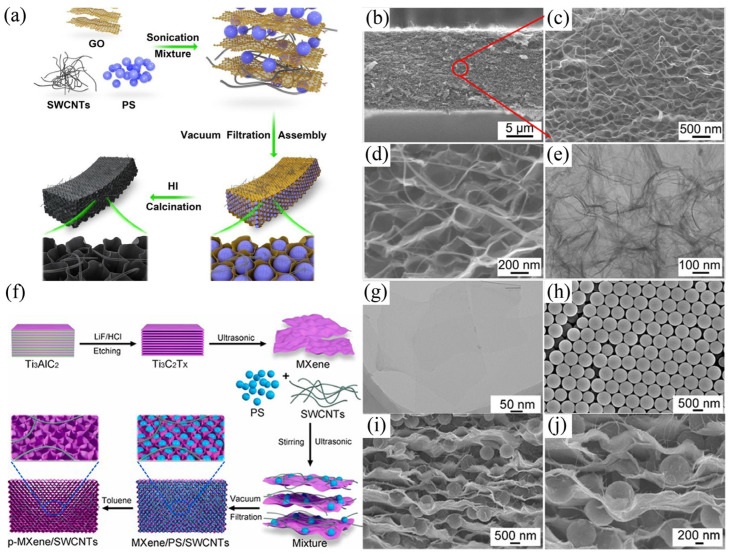
(**a**) The preparation schematic of rGO/SWCNTs film; (**b**–**d**) SEM images (cross-section) of the film; (**e**) TEM image of the film. Reprinted with permission from Reference [105]. Copyright 2022 American Chemical Society. (**f**) The preparation schematic of MXene/SWCNTs film; (**g**) TEM image of Mxene; (**h**) SEM image of PS microspheres (0.6) (0.6 means the diameter of PS spheres with 600 nm); (**i**,**j**) SEM images (cross-section) of MXene/PS/SWCNTs (0.6) composites. Reprinted from Reference [106] with permission.

Due to the demand of the agriculture, various herbicides and fertilizers used in this field have caused water pollution. In addition, methylene blue (MB), other colorants and urea phosphorus compounds are widely used in the printing and dyeing industry, and these organics will cause damage to the water environment. Therefore, the electrosorption method is also applied in the removal of various organic compounds in wastewater. Yue and co-workers reported a kind of rGO/SWCNTs film as the CDI electrode for removing MB (Figure 5a–e) [105]. For this system, PS was used as a template to introduce GO sheets for creating large pores and SWCNTs were distributed between the films for generating efficient pathways of ion diffusion. Consequently, the maximum adsorption capacity of rGO/SWCNTs film reached to 13,014.3 mg/g when applying −1.2 V voltage, and the capacity retention kept nearly 100% after five recycles. Furthermore, this group synthesized porous MXene/SWCNTs film as a CDI electrode for the removal of organic dyes in wastewater (Figure 5f–j), and the maximum adsorption capacity was as high as 28,403.7 mg/g when applying −2.4 V voltage [106]. In addition, our group prepared carbon foam electrodes derived from waste cigarette filters/zeolitic-imidazolate frameworks-8 (ZIF-8) composites [6,7,8,9,10,107], and found that the maximum electrosorption capacity of MB for these carbon foams reached to 1846.7 mg/g when applying −1.2 V voltage. Under most conditions, the adsorption time of methyl orange (MO) solution using most water treatment methods is more than 1 h. Whereas, Liu and co-workers used the holey graphene hydrogel (R-HGH) prepared by one-step hydrothermal method as the CDI electrode [108]. When 0.6 V voltage was applied to the R-HGH, the electrosorption capacity for 100 mg/L MO solution was 57 mg/g, and the adsorption equilibrium time could be within 200 s. Therefore, electrosorption can provide an effective route for the removal of typical organic compounds.

## 5. Summary and Prospects

In summary, CDI technology exhibits the advantages of low energy consumption, simple equipment, convenient operation, long cycle life, and environmental friendliness. It has a great development and potential application in the field of water treatment. However, the following problems restrict the wide application of CDI technology: (1) It lacks a comprehensive and reliable model to achieve reasonable economic optimization and reduce the investment cost. Additionally, the mechanism and model of electrosorption still need to be explored and studied. (2) The electrosorption technology still has some limitations such as short running cycle, poor stability, and low current efficiency.

Future studies need to optimize the cell architecture of CDI for making it more simple, convenient, and efficient. Table 1 lists the CDI performances of various carbon-based electrode materials reported in the literature. As seen, the deionization performance of CDI technology is not only related to the initial concentration of adsorbed solution, but also significantly influenced by the characteristics of electrode materials. However, poor EDL capacitance of carbon materials limits the electrosorption capacity. Compared with traditional carbon materials, pseudocapacitor electrode materials based on REDOX reaction have higher electrosorption adsorption capacity and fewer side reactions. Meanwhile, the increase in adsorption capacity can also reduce the electrode regeneration frequency. Therefore, the development of highly stable and low-cost pseudocapacitance materials is also the focus of CDI electrode materials in the future. In addition, multifunctional adsorption electrodes also will be the development trend for CDI system, which can selectively adsorb, separate and/or degrade the target materials efficiently. It is worth mentioning that green energy such as solar energy and wind energy can be integrated with CDI devices to improve the applicability of this technology.

Although CDI technology still have many challenges, we believe that this technology will have a broad application prospect with the further development of electric adsorption technology.

## Figures and Tables

**Table 1:** CDI performances of various carbon-based electrode materials.

Solution (mg/L)	Electrodes	Specific Surface Area (m^2^/g)	Electrical Conductivity	Applied Voltage (V)	Ion Capacity (mg/g)	Removal Efficiency (%)	Operation Time (h)	Ref.
NaCl (100)	ACF/rGO	649	42.6 (S/m)	1.2	9.2	-	0.5	[65]
	ACF	630	2.1 (S/m)	1.2	~4	-		
NaCl (300)	SWCNTs/rGO	308.37	Specific capacitance 36.35 F/g	2	48.73	-	10	[66]
	MWCNTs/rGO	262.44	-	2	39.53	-		
NaCl (50)	AGHZ *	-	Specific capacitance 746.5 F/g	1.2	9.95	83.65	2	[76]
NaCl (1500)	Co_3_O_4_@CNF@CNT	320.8	Specific capacitance 395 F/g	1.4	58.6	-	-	[79]
NaCl (200)	ACF/PANI	415	-	2	19.9	-	2	[87]
NaCl (500)	Graphene/carbon aerogel	546.2	-	-	26.9	-	10	[92]
	PANI/GO carbon aerogel	542.8	Specific capacitance 220 F/g	1.2	15.7	-	37 min	[93]
NaCl (1000)	Porous carbon nanoflake	408.1	Specific capacitance 187.6 F/g	1.2	16.29	-	0.5	[95]
Cu^2+^ (100)	PPy/CS/CNT composite	33.51	Specific capacitance 103.19 F/g	0.8	16.83	80.08	0.5	[86]
	WO3/PPy/ACF // ACF	788.27	Areal capacitance (2.58 F/cm^2^)	1	-	97.8	5	[88]
	Chitosan impregnated ACF cloth	1123.3	-	0.3	0.380 mmol/g	-	12	[100]
Cu (NO_3_)_2_ (-)	Two MnO_2_/CF electrodes	-	Specific capacitance 387 F/g	0.8	172.88	-	-	[103]
MB (30)	prGO/SWCNTs film	93.8	~74 (S/cm)	1.2	730.6	95	24	[105]
	p-MXene/SWCNTs film	42.9	-	1.2	1068.8	95	24	[106]
				0	55.8	-	24	
MB (-)	Carbon foam	1457	-	1.2	-	98	12	[107]
			-	0	-	63	24	

* ZnO nanoparticle-incorporated AC/graphene hydrogel composites.

## Data Availability

Not applicable.
